# Discovery of pathway-independent protein signatures associated with clinical outcome in human cancer cohorts

**DOI:** 10.1038/s41598-022-23693-w

**Published:** 2022-11-11

**Authors:** Mariam M. Konaté, Ming-Chung Li, Lisa M. McShane, Yingdong Zhao

**Affiliations:** Biometric Research Program, Division of Cancer Treatment and Diagnosis, National Cancer Institute, National Institutes of Health, Rockville, MD 20850 USA

**Keywords:** Cancer, Prognostic markers

## Abstract

Proteomic data provide a direct readout of protein function, thus constituting an information-rich resource for prognostic and predictive modeling. However, protein array data may not fully capture pathway activity due to the limited number of molecules and incomplete pathway coverage compared to other high-throughput technologies. For the present study, our aim was to improve clinical outcome prediction compared to published pathway-dependent prognostic signatures for The Cancer Genome Atlas (TCGA) cohorts using the least absolute shrinkage and selection operator (LASSO). RPPA data is particularly well-suited to the LASSO due to the relatively low number of predictors compared to larger genomic data matrices. Our approach selected predictors regardless of their pathway membership and optimally combined their RPPA measurements into a weighted risk score. Performance was assessed and compared to that of the published signatures using two unbiased approaches: 1) 10 iterations of threefold cross-validation for unbiased estimation of hazard ratio and difference in 5-year survival (by Kaplan–Meier method) between predictor-defined high and low risk groups; and 2) a permutation test to evaluate the statistical significance of the cross-validated log-rank statistic. Here, we demonstrate strong stratification of 445 renal clear cell carcinoma tumors from The Cancer Genome Atlas (TCGA) into high and low risk groups using LASSO regression on RPPA data. Median cross-validated difference in 5-year overall survival was 32.8%, compared to 25.2% using a published receptor tyrosine kinase (RTK) prognostic signature (median hazard ratios of 3.3 and 2.4, respectively). Applicability and performance of our approach was demonstrated in three additional TCGA cohorts: ovarian serous cystadenocarcinoma (OVCA), sarcoma (SARC), and cutaneous melanoma (SKCM). The data-driven LASSO-based approach is versatile and well-suited for discovery of new protein/disease associations.

## Introduction

Large-scale omics data characterizing human tumors can be leveraged to develop a deeper understanding of biological processes and predict clinical outcomes. For instance, one can develop prognostic molecular signatures to stratify patients into risk groups for disease progression or metastasis^1–3^. Multiple studies have demonstrated that molecular characterization of tumors may provide a more accurate and granular picture of a patient’s prognosis than the traditional pathological staging system, thus informing therapeutic and disease surveillance strategies^4–6^.

The Cancer Genome Atlas (TCGA) program has generated molecular profiles for thousands of human tumors spanning over thirty different tissue types^7^. Detailed genomic analyses using these data have identified novel cancer driver genes and biomarkers of disease^8–11^. To complement the genomic, epigenetic and transcript level data of TCGA, a more recent project by Akbani et al. has generated proteomic data from reverse-phase protein arrays (RPPA)^12^. RPPA is a high-throughput and cost-effective antibody-based method that provides a more direct assessment of cellular activity compared to DNA and RNA sequencing, which generate data that do not always correlate with protein expression^13^. Protein levels and post-translational modifications, such as phosphorylation and acetylation, are thought to better represent active pathway signaling.

Multiple studies have demonstrated the prognostic value of RPPA data^12,14–17^. Some of these studies have used pathway-driven approaches, relying on prior knowledge from the literature to group proteins into biological pathways, to develop prognostic signatures or predictors of treatment response. For instance, in the paper by Akbani et al*.* that introduced The Cancer Proteome Atlas (TCPA), proteins analyzed by RPPA were assigned to ten cancer-related pathways on the basis of a literature search of review articles on these pathways^12^. For a given pathway, positive regulatory elements of the pathway were assigned a coefficient of + 1. Correspondingly, the coefficient of negative regulatory elements of the pathway were set to − 1. Effectively, the pathway activity score was defined as the sum of positive regulators minus the sum of negative regulators of the pathway. This approach did yield pathway activity scores with prognostic value in some cancer types^12^. However, this approach may not be generally applicable as for many cancer types, involved pathways and regulator genes are not well defined^18^. We therefore hypothesized that a statistical approach specifically geared toward outcome prediction may yield scores with improved prognostic ability.

Using normalized RPPA data for up to 258 total, cleaved, acetylated, or phosphorylated proteins from TCPA^19,20^, we demonstrate the capability of a statistical approach, LASSO regression^21^, to derive weighted risk scores that achieve strong prognostic stratification without requiring a priori biological knowledge. Unbiased statistical resampling methods were applied to proteomic data from four TCGA cancer studies to demonstrate that performance of our LASSO-based prognostic scores is equivalent or superior to that of predefined pathway-driven RPPA signatures.

## Results

### Three-fold cross-validation model assessment

**Figure 1:**
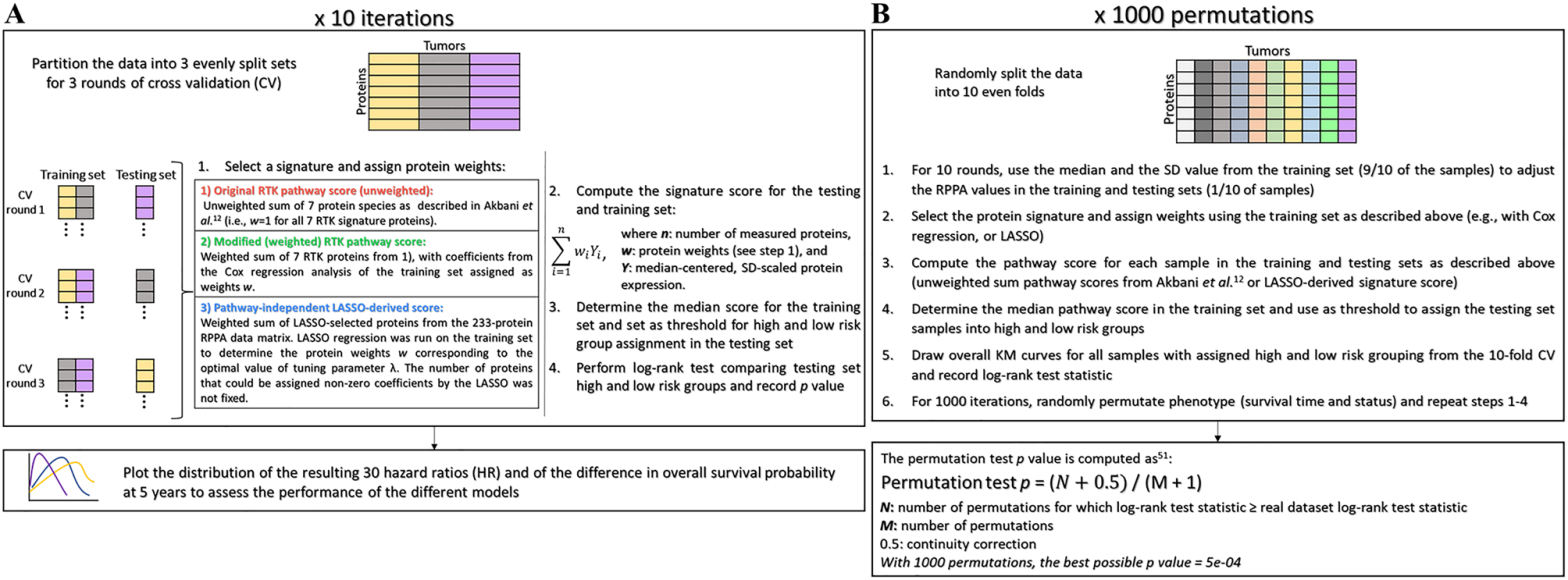
Schematic representations of the unbiased model evaluation approaches. (**A**) Ten iterations of threefold cross-validation. (**B**) Permutation test with 1000 permutations.

The number of samples in the KIRC dataset was comparable between the version of TCPA that we downloaded for our analysis, and the version used in the original study by Akbani et al*.*(Table 1)^12^. We first repeated the Kaplan–Meier analysis of the KIRC dataset with the modifications noted in the methods and illustrated in Fig. 1A: for ten iterations, we split the dataset into three folds and assigned tumors to a training set (2/3) and a testing set (1/3). The training set median and s.d. were used to adjust RPPA values in all 445 tumors. Subsequently, the unweighted RTK signature score was computed for all tumors, and testing set tumors were assigned into high and low risk group based on median RTK score in the training set. The resulting thirty pairs of high risk and low risk Kaplan–Meier curves are displayed in Supplementary Fig. S1. Then, Cox regression weighted RTK pathway scores and LASSO-regression derived protein signature scores were evaluated following the same procedure. The resulting Kaplan–Meier curves are shown in Supplementary Fig. S2 and Supplementary Fig. S3, respectively.

**Table 1:** Data summary.

TCGA tissue code	Tissue	Samples in Akbani et al*.*^12^	Samples in TCPA 07/18/18 release	Difference
*BLCA*	Bladder cancer	127	344	+ 217
*BRCA*	Breast cancer	747	874	+ 127
*COADREAD*	Colorectal	464	487	+ 23
*GBM*	Glioblastoma	215	205	− 10
*HNSC*	Head & neck cancer	212	346	+ 134
*KIRC*	Kidney clear cell carcinoma	454	445	− 9
*LUAD*	Lung adenocarcinoma	237	362	+ 125
*LUSC*	Lung squamous cell carcinoma	195	325	+ 130
*OVCA*	Ovarian serous cystadenocarcinoma	412	411	− 1
*SARC*	Sarcoma	0	216	+ 216
*SKCM*	Skin cutaneous melanoma	0	315	+ 315
*UCEC*	Endometroid cancer	404	404	0

Number of tumor samples included in the original analysis^12^ compared to the number of samples in the present study and downloaded from MD Anderson Cancer Center’s The Cancer Proteome Atlas TCPA^19,20^.

**Figure 2:**
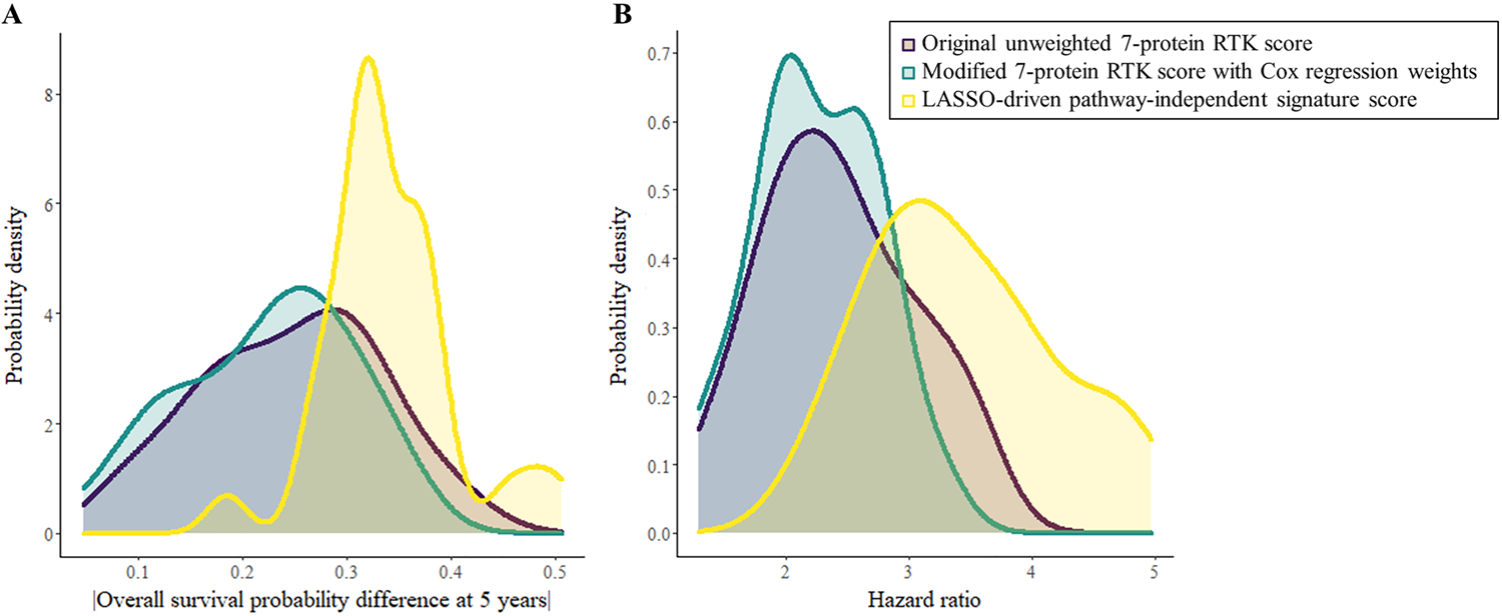
Probability density distribution of (**A**) difference in overall survival probability at 5 years, and (**B**) hazard ratio for high vs. low risk TCGA-KIRC groups stratified according to the original RTK score, LASSO-modified RTK score, Cox regression-modified RTK score, or pathway independent LASSO-derived signature score.

Re-assigning weights to the 7 RTK proteins based on Cox regression did not improve model performance compared to the original, unweighted RTK score; however, deriving a new, pathway-independent LASSO-driven score improved the stratification of patients into high and low risk groups. Median difference in overall survival probability at 5 years based on the LASSO-derived risk score was 32.8%, compared to 25.2% when using the 7-protein unweighted RTK score (Fig. 2A). Median hazard ratio (HR) between high and low risk groups across the held-out folds in the CV based on the 7-protein RTK score was 2.4, compared to 3.3 when using the risk score derived by LASSO applied to the training data folds (Fig. 2B). Time-dependent ROC curves for overall survival probability at 5 years for all three prognostic models are shown in Supplementary Fig. S4A–C. Boxplots of risk scores stratified by pathologic stage for all three types of risk scores in KIRC revealed a weak linear trend in association between risk score and stage (Supplementary Fig. S5A–C).

### Permutation test for the evaluation of the cross-validated log-rank statistic

**Figure 3:**
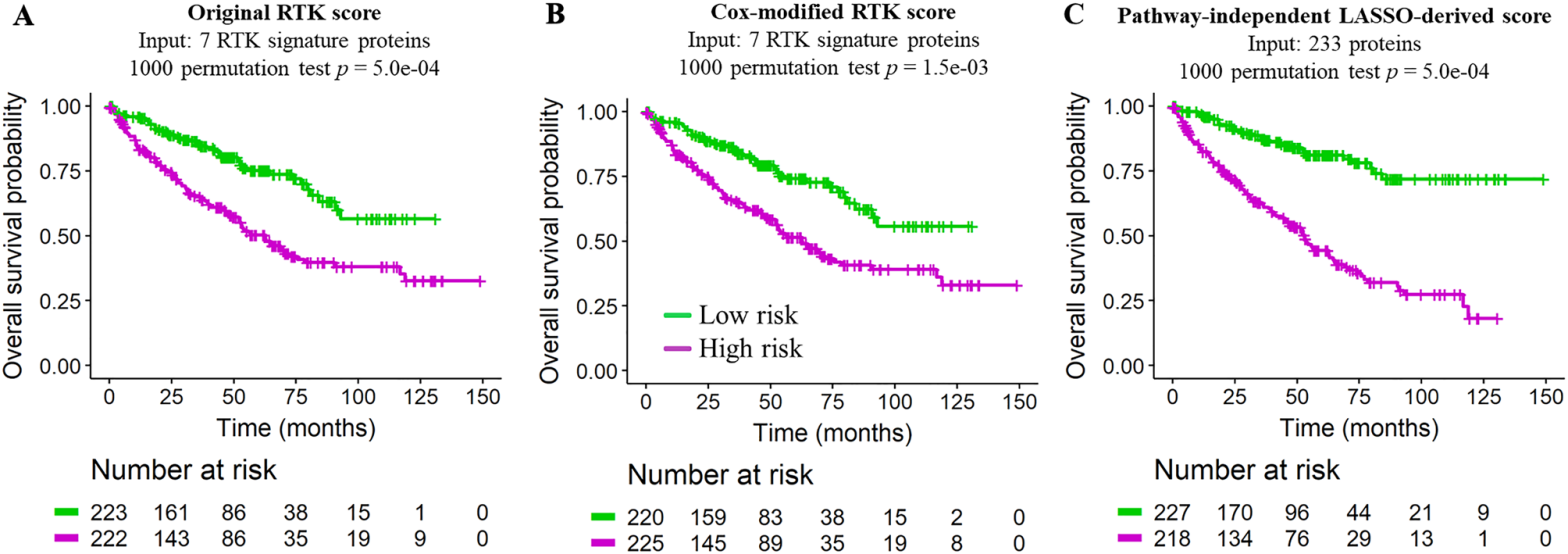
Kaplan–Meier overall survival analysis of KIRC samples stratified according to different signature scores. Kaplan–Meier curves demonstrating the stratification of TCGA-KIRC tumors according to (**A**) the original 7-protein RTK score, (**B**) the COX regression weighted 7-protein RTK score, and (**C**) the pathway-independent LASSO-derived prognostic signature score. The high and low risk group curves are in purple and green, respectively.

As described in the Methods and schematized in Fig. 1B, for each of the three prognostic models (unweighted RTK pathway score, Cox regression weighted RTK score, and LASSO-derived protein signature score), the statistical significance of the cross-validated log-rank statistic was evaluated with a 1000 permutation test^22^. Tumor stratification based on the original RTK score or on the pathway-independent LASSO-derived score obtained the best possible permutation test *p* value after 1000 permutations (i.e. permutation test *p* = 5e − 04); however, the split between high and low risk groups was more pronounced with the LASSO-derived pathway-independent score (Fig. 3A,C). The Cox regression weighted RTK pathway score method resulted in a somewhat larger, but still statistically significant, permutation test *p* value = 1.5e − 03 (Fig. 3B).

Stage-separated and sex-separated Kaplan–Meier curves for the three types of risk scores (original RTK score, Cox-modified RTK score, and LASSO-derived score) in KIRC were also generated (Supplementary Fig. S6). A visual examination reveals that the performance of the risk scores is independent of sex, and even at lower pathologic stages, the LASSO-derived risk score effectively stratified patients into better and worse prognoses (Supplementary Fig. S6C). In contrast, the 7 protein RTK score from Akbani et al*.* whether in its original form or modified with Cox regression coefficients, performed worse for the stratification of low stage tumors (Supplementary Fig. S6A–B).

The top 20 proteins most frequently selected by the LASSO are listed in Table 2. Multiple proteins have previously been implicated in kidney cancer^14,23–26^, and interestingly, 13 of these 20 proteins were not assigned to any of the ten cancer-related pathways in the original paper by Akbani et al*.*^12^. The remaining 7 proteins were annotated as belonging to different pathways (TSC_mTOR, Hormone_b, Cell_cycle, Ras_MAPK, and DNA_damage_response), none of which were the RTK pathway (Supplementary Table S1). Furthermore, except for MAPK_pT202_Y204, the expression of the top 20 proteins did not strongly correlate to that of the 7 RTK proteins from the original prognostic signature (Supplementary Table S2). These results provide support for the use of a pathway-independent method to optimize the selection of prognostic protein markers from the RPPA data matrix.

**Table 2:** Top 20 proteins most frequently selected by the LASSO and pathway assignment from reference^12^ (Supplementary Table S1).

Protein marker ID	Gene	Name	Pathway assignment	References in RCC
*4EBP1_pT37_T46*	EIF4EBP1	Eukaryotic translation initiation factor 4E-binding protein 1	TSC_mTOR	^23,24^
*ACC1*	ACACA	Acetyl-CoA carboxylase 1	NA	^14,25^
*AMPK-alpha_pT172*	PRKAA1	5′-AMP-activated protein kinase catalytic subunit alpha-1	NA	^26^
*AR*	AR	Androgen receptor	Hormone_b	^27^
*A-Raf_pS299*	ARAF	Serine/threonine-protein kinase A-Raf	Ras_MAPK	
*B-Raf_pS445*	BRAF	Serine/threonine-protein kinase B-raf	NA	
*Caveolin-1*	CAV1	Caveolin-1	NA	^28^
*CDK1*	CDK1	Cyclin-dependent kinase 1	Cell_cycle	^29^
*c-Myc*	MYC	Myc proto-oncogene protein	NA	^30^
*Gab2*	GAB2	GRB2-associated-binding protein 2	NA	
*IGFBP2*	IGFBP2	Insulin-like growth factor-binding protein 2	NA	
*MAPK_pT202_Y204*	MAPK1, MAPK3	Mitogen-activated protein kinase 1/3	Ras_MAPK	
*MIG6*	ERRFI1	ERBB receptor feedback inhibitor 1	NA	
*p70-S6K_pT389*	RPS6KB1	Ribosomal protein S6 kinase beta-1	TSC_mTOR	^31^
*PEA-15*	PEA15	Astrocytic phosphoprotein PEA-15	NA	^32^
*Rad51*	RAD51	DNA repair protein RAD51 homolog 1	DNA_damage_response	^14,33^
*SCD1*	SCD	Stearoyl-CoA desaturase	NA	^34^
*SF2*	SRSF1	Serine/arginine-rich splicing factor 1	NA	^35^
*Stat3_pY705*	STAT3	Signal transducer and activator of transcription 3	NA	^36^
*Syk*	SYK	Tyrosine-protein kinase SYK	NA	

Literature references associating these proteins with patient prognosis in renal clear cell carcinoma (RCC) are also listed.

### LASSO-derived RPPA scores have prognostic value in other tumor types represented in TCGA

To assess whether our proposed LASSO-derived approach yields scores with prognostic value in other human tumor datasets, we compared the performance of ten literature-driven pathway scores to that of the purely statistical LASSO-derived protein signature score in 3 additional datasets from TCGA: 353 skin cutaneous melanomas (SKCM), 221 sarcomas (SARC), and 411 ovarian serous cystadenocarcinoma (OVCA). Clinical characteristics of the datasets are detailed in Table 3.

**Table 3:** Clinical and protein characteristics of the TCGA datasets evaluated in the permutation test.

TCGA cohort	KIRC	OVCA	SARC	SKCM
Number of patients	445	406	216	315
**Sex, n (%)**				
Female	148 (33.3%)	406 (100%)	113 (52.3%)	127 (40.3%)
Male	297 (66.7%)	0 (0%)	103 (47.7%)	188 (59.7%)
**Pathologic stage, n (%)**				
Stage I	216 (48.5%)	13 (3.2%)	NA	49 (15.6%)
Stage II	44 (9.9%)	21 (5.2%)	NA	78 (24.8%)
Stage III	108 (24.3%)	317 (78.1%)	NA	128 (40.6%)
Stage IV	76 (17.1%)	52 (12.8%)	NA	20 (6.3%)
Stage X (i.e. NA)	1 (0.2%)	3 (0.7%)	NA	40 (12.7%)
**Overall survival status, n (%)**				
Living	288 (64.7%)	161 (39.7%)	134 (62.0%)	165 (52.4%)
Deceased	157 (35.3%)	245 (60.3%)	82 (38.0%)	150 (47.6%)
Follow-up (months)	0.1–149.1	0.3–180.1	0.5–171.1	0.2–369.9
**Age (years)**				
Range	26–90	26–89	20–90	15–90
Median	61	58	62	57
**LASSO regression**				
Number of proteins measured	233	211	217	216
Number of non-zero coefficients	25	22	15	20

*NA* not applicable or unknown, *KIRC* kidney clear cell carcinoma, *OVCA* ovarian serous cystadenocarcinoma, *SARC* sarcoma, *SKCM* skin cutaneous melanoma.

Representative plots of the cross-validated optimization of the regularization parameter λ on the three datasets and non-zero coefficients assigned by the LASSO are shown in Supplementary Fig. S7. Boxplots of LASSO-derived risk scores stratified by pathologic stage presented in Supplementary Fig. S5D–E demonstrate that in the OVCA and SKCM datasets, there is little to no association between risk score and tumor stage.

**Figure 4:**
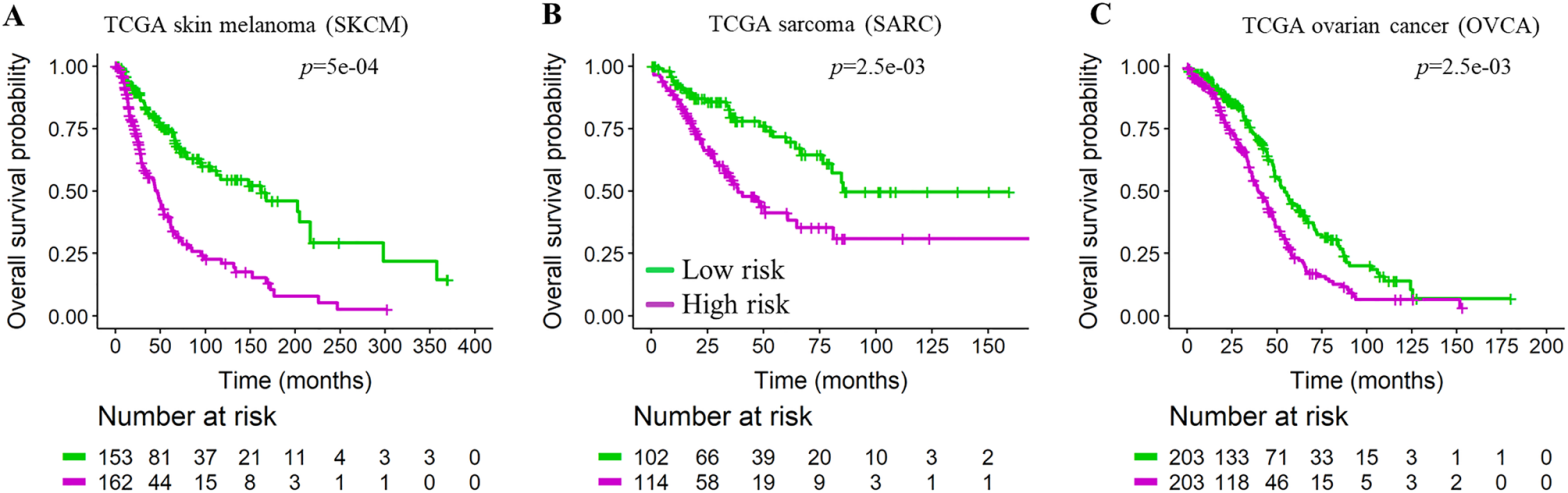
Kaplan–Meier curves demonstrating the stratification of tumors from TCGA according to the pathway-independent LASSO-derived prognostic signature score for multiple tumor types: (**A**) skin melanoma, (**B**) sarcoma, and (**C**) ovarian carcinoma. Permutation test *p* values are shown. The high and low risk group curves are in purple and green, respectively. Published pathway-specific unweighted signatures introduced by Akbani et al*.*^12^ were also evaluated for comparison (see Table 4).

The performance of the different scoring methods was evaluated with a 1000 permutation test, as for KIRC. The resulting cross-validated Kaplan–Meier curves for high and low LASSO-derived risk scores for these three datasets demonstrate the statistically significant stratification of the tumors into high and low risk groups (Fig. 4A–C).

Stage separated Kaplan–Meier curves were plotted for OVCA and SKCM, and sex separated curves were plotted for SKCM and SARC (Supplementary Fig. S8). The SARC dataset in TCGA did not have any pathologic stage nor tumor grade information and the OVCA dataset only contains female patients. In OVCA, the vast majority of tumors are stage III (78%, see Table 3), hence the visible difference in survival probability between high and low score stage III tumors (Supplementary Fig. S8A). The very low sample size and low number of events in the lower stages (stage I and II tumors together account for ~ 8% of the dataset) make the corresponding Kaplan–Meier curves less compelling. In SKCM, high and low score effectively align with patient survival (Supplementary Fig. S8B). In these datasets as well, performance of risk scores was independent of sex (Supplementary Fig. S8B–C).

Furthermore, permutation test *p* values for pathway-^12^ or LASSO-driven protein signature in the three TCGA studies are listed in Table 4. In SKCM and SARC, our LASSO-based approach performed consistently well and yielded smaller *p*-values than all ten literature-curated unweighted pathway scores (*p* = 5e − 04). In OVCA, the *p*-value for the LASSO-derived protein signature score was only matched by that of the Ras-MAPK pathway score (*p* = 2.5e − 03). For SKCM and SARC, the LASSO-derived signatures mostly contained proteins that did not have a pre-defined pathway assignment in the original study^12^ (Supplementary Fig. S7A–B). Moreover, for OVCA, the LASSO-derived signature was composed of 13 proteins that did not belong to any of the ten pre-defined pathways from Akbani et al*.* and nine proteins belonging to eight of the pre-defined pathways (Supplemental Fig. S7C). Taken together, these results suggest that more than one pathway may inform prognosis, thus placing pathway-specific approaches at a disadvantage for prognostic modeling.

**Table 4:**
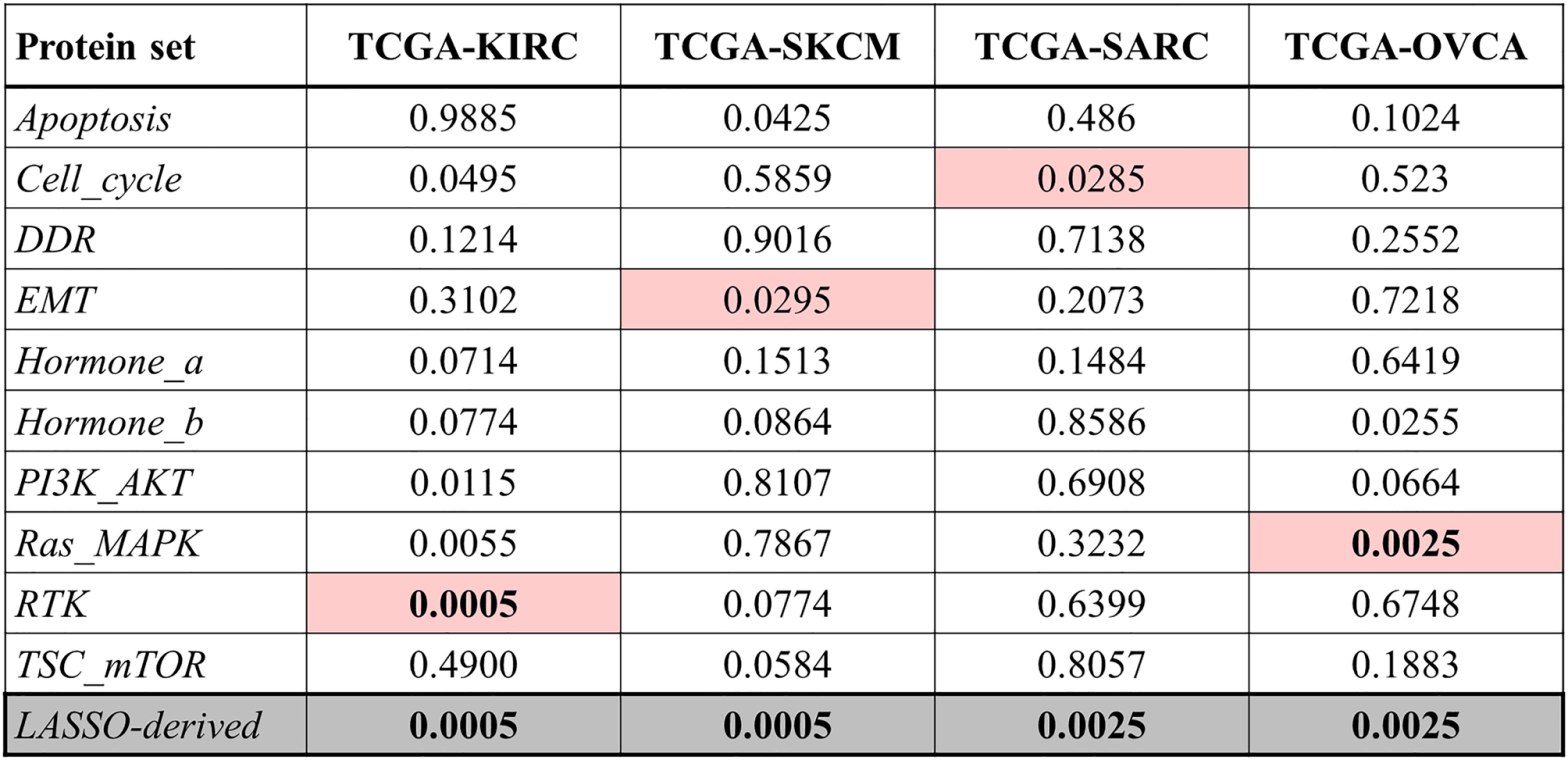
Permutation test *p* values for pathway-^12^ or LASSO-driven protein signature in TCGA studies.

Scores were computed as described above: unweighted sums of pathway member proteins for pre-defined signatures from Akbani et al*.* and weighted sum of LASSO-selected proteins. Most significant permutation test *p* values in the pathway-derived approach are indicated for each dataset with red highlights to facilitate the comparison with the performance of the LASSO method (gray highlight). In each column, the most significant *p* value is in bold font.

KIRC kidney clear cell carcinoma, OVCA ovarian serous cystadenocarcinoma, SARC sarcoma, SKCM skin cutaneous melanoma.

## Discussion

Assessing the functional proteome via the analysis of RPPA data may yield important insights into patient prognosis and therapy options. We used two unbiased statistical approaches to compare the performance of our pathway-independent LASSO-derived method to that of a predefined pathway-driven risk score (Fig. 1A,B). We found our LASSO-derived method for the selection of a data-driven prognostic signature to be effective for the stratification of patient samples into high and low survival risk groups (Supplementary Fig. S3 and Supplementary Fig. S4C). Our LASSO-based approach to derive a prognostic signature performed as well or better than a biology-driven prognostic signature for the TCGA kidney clear cell carcinoma dataset according to both unbiased evaluation approaches (Figs. 2A,B, 3A–C, and Supplementary Fig. 4A–C). Our method was successfully applied to three other TCGA cancer studies in which it performed as well or better than predefined pathway-driven RPPA signatures (Fig. 4A–C).

Pathway-based approaches have limitations and are susceptible to biases depending on which molecules are included from a given pathway. They require prior knowledge of pathways and regulators of the cancer type under study. Mubeen et al*.* justly noted that different pathway databases contain different representations of the same biological pathway^37^. Correspondingly, they found that the choice of pathway database for statistical enrichment analysis or predictive modeling had a profound impact on results. Another recent study by Chen et al*.* came to the same conclusion^38^. Moreover, cancer is an extremely complex disease often involving the concerted dysregulation of multiple pathways^39^. Therefore, using a single literature-defined pathway for prognostic prediction runs the risk of overlooking informative molecules assigned to a different pathway. Indeed, in the TCGA datasets examined for the present study, the majority of proteins most frequently selected by the LASSO were not assigned to any of the 10 cancer-associated pathways curated by Akbani et al*.* (Table 2 and Supplementary Fig. S7)^12^. For KIRC, only 7 out of the top 20 most frequently selected proteins overlapped with one or more of the 10 predefined pathways from Akbani et al. The analysis of SKCM, SARC, and OVCA also revealed that the majority of LASSO-selected predictors were not in the pathways defined by Akbani et al*.* despite being assigned strong weights by the LASSO, and belong to a wide variety of cancer-associated pathways such as the Hippo pathway (e.g. YAP, TAZ) and inflammatory immune response (e.g. PDL1, NFKBP65_pS536) (Supplementary Fig. S7), consistent with the widespread dysregulation that is typical of cancer^40^.

In our study, LASSO regression on the KIRC RPPA dataset consistently yielded signatures including proteins which have previously been linked to survival in kidney cancer specimens (Table 2). For instance, AMPK is a sensor of cellular energy and negative regulator of the mTOR signaling pathway ^26^. Foersch et al*.* demonstrated the significant association between androgen receptor (AR) and prognosis in patients with renal clear cell carcinoma (RCC)^27^. Cytoplasmic CAV1 protein expression measured by immunohistochemistry (IHC) was found to correlate with clinical prognosis is RCC^28^. CDK1 and CDK2 activity was linked to poor prognosis and RCC recurrence^29^. Bellut et al*.* showed that c-MYC protein expression had prognostic value in a subtype of RCC^30^. The phosphorylation of ribosomal protein S6 kinase beta-1 (p70S6K) is a downstream target of mTOR and confirmed prognostic marker in RCC^31^. SF2, a novel oncoprotein in RCC, was significantly associated with poor survival in a large cohort of patients with RCC^35^. High SCD1 expression was prognostic of overall survival in patients with RCC^34^. Nuclear expression of p-STAT3 was significantly associated with RCC subtypes with greater malignant potential^36^. 4E-BP1, a regulator of mRNA translation initiation, is activated by mTORC1 signaling in response to extracellular stimuli and metabolic stress conditions^41^. A recent study by Naito et al*.* revealed an association between 4EBP1 phosphorylation and poor prognosis in a non-metastatic cohort of renal clear cell carcinoma (RCC)^23^. Correspondingly, Campbell et al*.* had demonstrated that the combined expression of p4E-BP1 and eIF4E was associated with significantly worse disease-free survival in patients with RCC^24^. Furthermore, acetyl-CoA carboxylase (ACC1) was also systematically selected by the LASSO (Table 2). A defining feature of KIRC is the presence of lipid and glycogen-rich cytoplasmic deposits ^25^. Du et al*.* identified hypoxia-inducible factor (HIF) control of fatty acid metabolism as being essential for KIRC tumorigenesis. ACC1 carries out a major step of fatty acid synthesis for membrane synthesis, production of energy stores and signaling molecules^42^. Interestingly, the expression of lipogenic enzymes including FASN, ACC1, and ACLY is also downstream of mTORC1 signaling^43^. Han et al*.* also reported the prognostic utility of ACC1 protein expression in KIRC, as well as FASN, Cyclin B1 and Rad51, which was also frequently selected by the LASSO in our study (Table 2)^14^.

The 258 proteins included in the RPPA for TCPA were selected on the basis of their functional role in cancer-related pathways such as proliferation, DNA damage, EMT, and apoptosis^12^. This focused approach confers an advantage for LASSO feature selection over the use of whole genome RNA-seq datasets which contain tens of thousands of genes, thus making the feature selection process highly susceptible to noise. Kim and Bredel reported similar findings in their 2013 publication^44^. The authors used gene expression profiles from 300 cancer pathway genes obtained from the Molecular Signature Database (MSigDb) and the Kyoto Encyclopedia of Genes and Genomes dataset (KEGG) as an input for LASSO optimization. They demonstrated that the gene pre-selection increased the average correlation coefficient between observed survival days and relate risks compared to the same analysis conducted on whole genome gene expression profiles^44^.

The data-driven nature of our LASSO-based approach makes it versatile and particularly well-suited for the discovery of unexplored protein/disease associations that could aid in therapeutic discovery.

## Methods

### Data acquisition

Level 4, batch-corrected proteomic data generated by reverse phase protein array (RPPA) for up to 258 total, cleaved, acetylated, or phosphorylated proteins across 7694 patient tumors were obtained from The Cancer Proteome Atlas (TCPA) data portal (https://tcpaportal.org/tcpa/) version 4.2 (release date: 07/18/2018)^19,20^. The tumors included 445 kidney clear cell carcinomas (KIRC), 353 skin cutaneous melanomas (SKCM), 221 sarcomas (SARC), and 411 ovarian serous cystadenocarcinoma (OVCA). Survival data, sex, and pathologic stage information for the patient tumors were downloaded from the Broad Institute’s cBioPortal for Cancer Genomics^45,46^, and were matched to the proteomic data by specimen ID. Table 1 summarizes the different tissue datasets downloaded from TCPA and compares the number of samples in our study to the number of samples used in the paper by Akbani et al*.*^12^.

For cross-validation steps described below, level 4 RPPA values downloaded from TCPA were median-centered and standard deviation (s.d.) normalized across tumors using the median protein expression and s.d. from each training set to yield relative protein expression levels in the testing set as described previously by Akbani et al.^12^.

### Unweighted RTK pathway score

The starting point of our study was a published RPPA-based seven-protein signature of receptor tyrosine kinase (RTK) pathway activity in the form of an unweighted sum of seven protein measurements: EGFR-pY1068, EGFR-pY1173, HER2-pY1248, HER3-pY1289, SHC-pY317, SRC-pY416, and SRC-pY527^12^. The prognostic value of this signature had been demonstrated by Akbani et al*.* in a 445-patient renal clear cell carcinoma cohort (TCGA-KIRC) ^12^. When computing the literature-driven, unweighted pathway score from Akbani et al. the protein weights ***w*** were assigned the value of + 1 or − 1. The pre-defined pathway members and weights are listed in Supplemental Table S1.

### Weighted RTK pathway score with Cox regression weights

Subsequently, we modified the original RTK score using Cox regression to derive new protein weights ***w*** for the seven proteins of the original RTK signature using R package *survival* (version 3.3-1)^47^. Cox regression was run on each training set within the cross-validation procedure, as described below, to optimize protein weights ***w*** for the seven proteins members of the RTK pathway according to the literature search conducted by Akbani et al*.*^12^. Subsequently, the protein signature score for each tumor was computed using the following equation:

1 $$\text{Protein signature score = }\sum_{i=1}^{n}{w}_{i}{Y}_{i}\text{,}$$

where ***n*** is the number of proteins with measurements, ***w*** is the vector of protein weights, and ***Y*** is the median-centered, SD-scaled protein expression matrix.

### LASSO-derived protein signature score

Finally, we derived a pathway independent protein signature score using LASSO regression with L_1_-penalty to select an unrestricted number of elements from the 233 proteins with RPPA measurements in this dataset, and optimally combine their RPPA measurements into a weighted risk score for the 445 KIRC tumors. LASSO regression was performed on each training set within the cross-validation procedure, as described below, to determine protein weights ***w*** corresponding to the optimal value of the tuning parameter λ using R package *glmnet* (version 4.1-4)^48^. Protein signature score was computed for all tumors using Eq. (1) as described above.

### Method performance evaluation

Because model building from a large number of candidate variables is prone to overfitting, we utilized two unbiased approaches for evaluation of method performance: (1) ten iterations of threefold cross-validation for unbiased estimation of hazard ratio and difference in 5-year survival (by Kaplan–Meier method) between high and low risk groups defined based on application of a median cut to the risk score; and (2) a permutation test to evaluate the statistical significance of the cross-validated log-rank statistic.

### Cross-validation

The prognostic scores developed using the Cox regression and LASSO approaches, and corresponding low and high risk groups defined by median cut, were first evaluated with ten iterations of three-fold cross-validation. R package *caret* (version 6.0-93) was used to split the dataset into folds for the cross-validation^49^. In order to test model stability, we used a different random seed for each of the ten iterations. The evaluation approach is illustrated in Fig. 1A. For each of the ten iterations, the dataset of 233 RPPA measurements for 445 KIRC tumors was randomly split into a training set (2/3 of the tumors) and a testing set (remaining 1/3 of the tumors) for three rounds of cross-validation (CV). At each CV round, the pathway score was computed on the training set and applied to all tumors as described above. Then, the median pathway score for the tumors of the training set was used as a stratification cutoff for high and low risk groups in the testing set. We then performed a log-rank test comparing testing set high and low risk groups using R package *survival*^47^ and recorded the log-rank test statistic. Hazard ratios and difference in overall survival probabilities at five years between high and low risk groups in the cross-validation testing set by Kaplan–Meier method were also documented. Time-dependent receiver operating characteristic (ROC) analysis was conducted using R package *survivalROC* (version 1.0.3) which implements the cumulative case/dynamic control ROC^50^. ROC for overall survival at 5 years (i.e. 60 months) was evaluated because in this dataset, > 70% of events had occurred by that time point.

### Assessment of model performance with the permutation test

As schematized in Fig. 1B, the dataset of 233 RPPA measurements for 445 KIRC tumors was randomly split into ten evenly-sized folds using R package *caret*^49^. For ten rounds, nine tenths of the data served as the training set, while the remaining tenth was assigned to the testing set. The resulting ten partitions were found to have similar pathologic stage and sex proportions to the complete dataset. For the unweighted RTK signature all seven protein weights were assigned the value of + 1. For the Cox regression weighted RTK signature and the LASSO-derived protein signature score, protein weights ***w*** were derived from the training set as described above. Protein signature scores were computed for all 445 tumors using Eq. (1). The median pathway or protein signature score in the training set was used as the threshold to assign the testing set tumors to high and low risk score groups. After the tenth round, with all 445 tumors having been assigned a high or low risk label, we drew the overall cross-validated Kaplan–Meier curves and recorded the log-rank test statistic for the original data. Then, for 1000 permutations, we randomly permuted the correspondence of phenotype (i.e. survival time and status) and protein expression, repeated the tenfold cross-validation, and computed the log-rank statistic. The permutation test *p* value was computed using the following equation described by Royston and Parmar^51^:

2 $$\text{Permutation test }{{p}}\text{ = }\frac{N+0.5}{M+1},$$

where ***N*** is the number of permutations for which log-rank test statistic was greater than or equal to the real dataset log-rank test statistic, ***M*** is the number of permutation (i.e. 1000), and 0.5 corresponds to the continuity correction constant. With 1000 permutations, the best possible permutation test *p* value = 5e − 04.

### Application to other TCGA cohorts

To test the broader applicability of our LASSO-based signature development approach, we selected three other TCGA studies—skin cutaneous melanomas (SKCM), sarcomas (SARC), and ovarian serous cystadenocarcinoma (OVCA)—and compared the resulting log-rank statistic for the LASSO-based patient stratification to that based on published unweighted pathway-driven protein signatures^12^. For each of the three datasets, we computed unweighted pathway scores for the 10 literature-curated pathways listed in Supplementary Table S1 and evaluated the model performances using the permutation test with 1000 permutations as was done for KIRC. LASSO-derived protein signature scores were derived as described for KIRC and were evaluated using the 1000-permutation test.

## Supplementary Information

Supplementary Information.

## Data Availability

R codes are available upon request. The datasets used for analysis are publicly available from TCGA Research Network (http://cancergenome.nih.gov/) and TCPA (https://tcpaportal.org/tcpa/download.html).
