# Supplementary material for: Discovery of pathway-independent protein signatures associated with clinical outcome in human cancer cohorts
Source: Sci Rep. 2022 Nov 11;12:19283. doi: 10.1038/s41598-022-23693-w (PMC9652455; doi:10.1038/s41598-022-23693-w)
Supplement: Supplementary file 1 — Supplementary Information. [file 41598_2022_23693_MOESM1_ESM.pdf]

# Discovery of pathway-independent protein signatures associated with clinical outcome in human cancer cohorts

**Mariam M. Konaté*, Ming-Chung Li, Lisa M. McShane, Yingdong Zhao**

**Supplementary Materials:**

- **Supplementary Figure S1**
- **Supplementary Figure S2**
- **Supplementary Figure S3**
- **Supplementary Figure S4**
- **Supplementary Figure S5**
- **Supplementary Figure S6**
- **Supplementary Figure S7**
- **Supplementary Figure S8**
- **Supplementary Table S1**
- **Supplementary Table S2**

**Supplementary Figure S1.** Kaplan-Meier curves for 10 iterations of 3-fold cross validation of unweighted RTK pathway score in TCGA-KIRC. The high and low risk group curves are in purple and green, respectively.

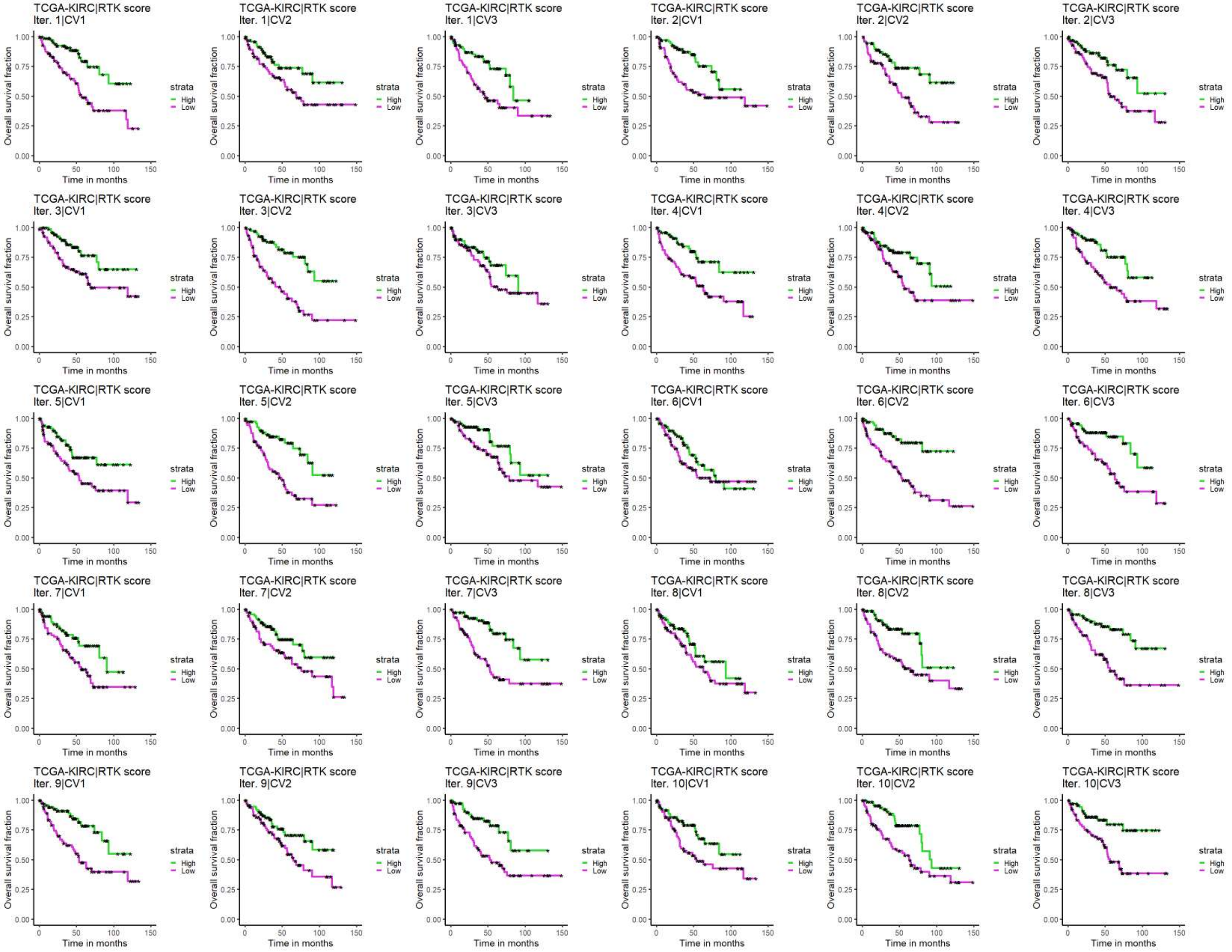

**Supplementary Figure S2.** Kaplan-Meier curves for 10 iterations of 3-fold cross validation of Cox regression weighted RTK pathway score in TCGA-KIRC. The high and low risk group curves are in purple and green, respectively.

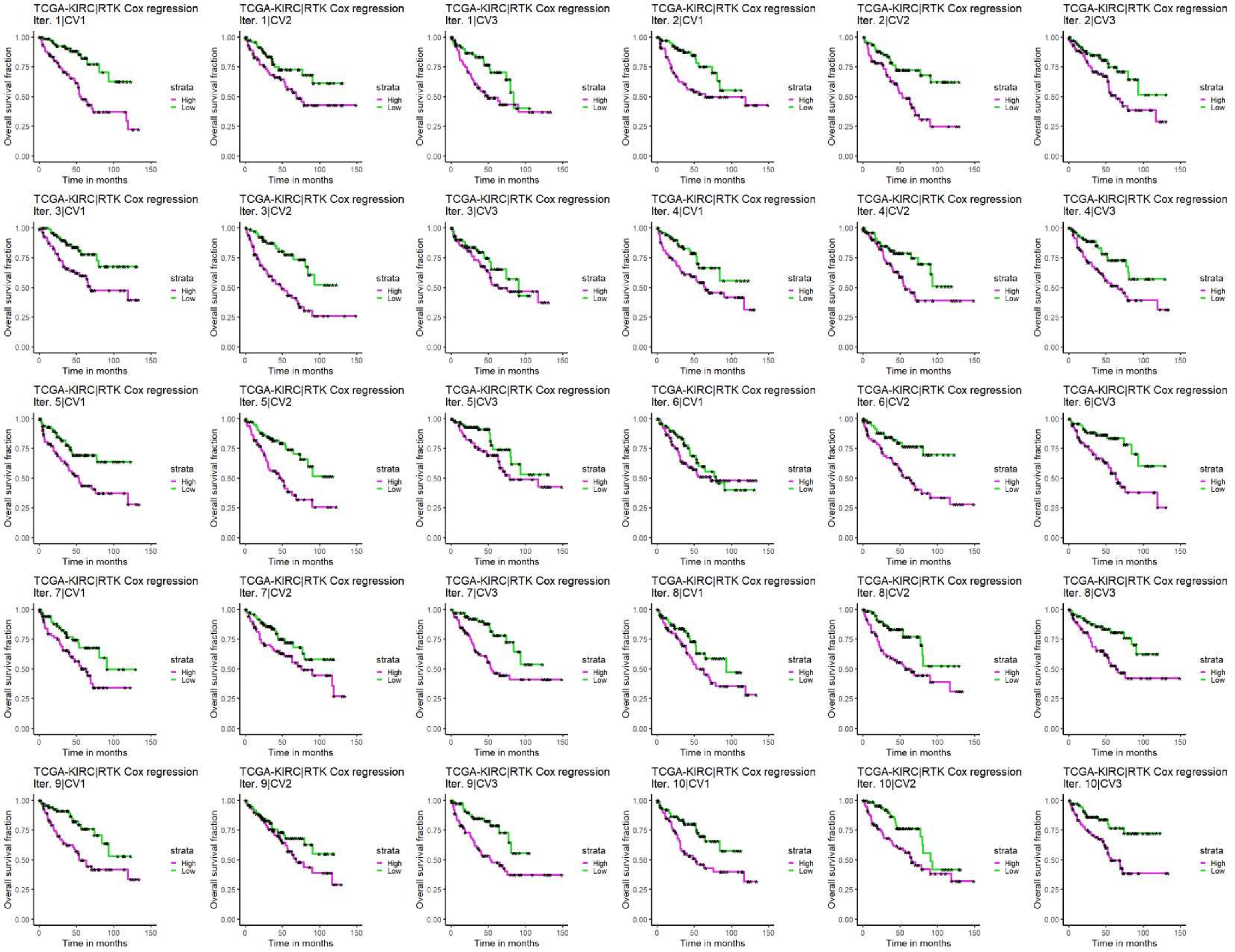

**Supplementary Figure S3.** Kaplan-Meier curves for 10 iterations of 3-fold cross validation of LASSO-derived protein signature score in TCGA-KIRC. The high and low risk group curves are in purple and green, respectively.

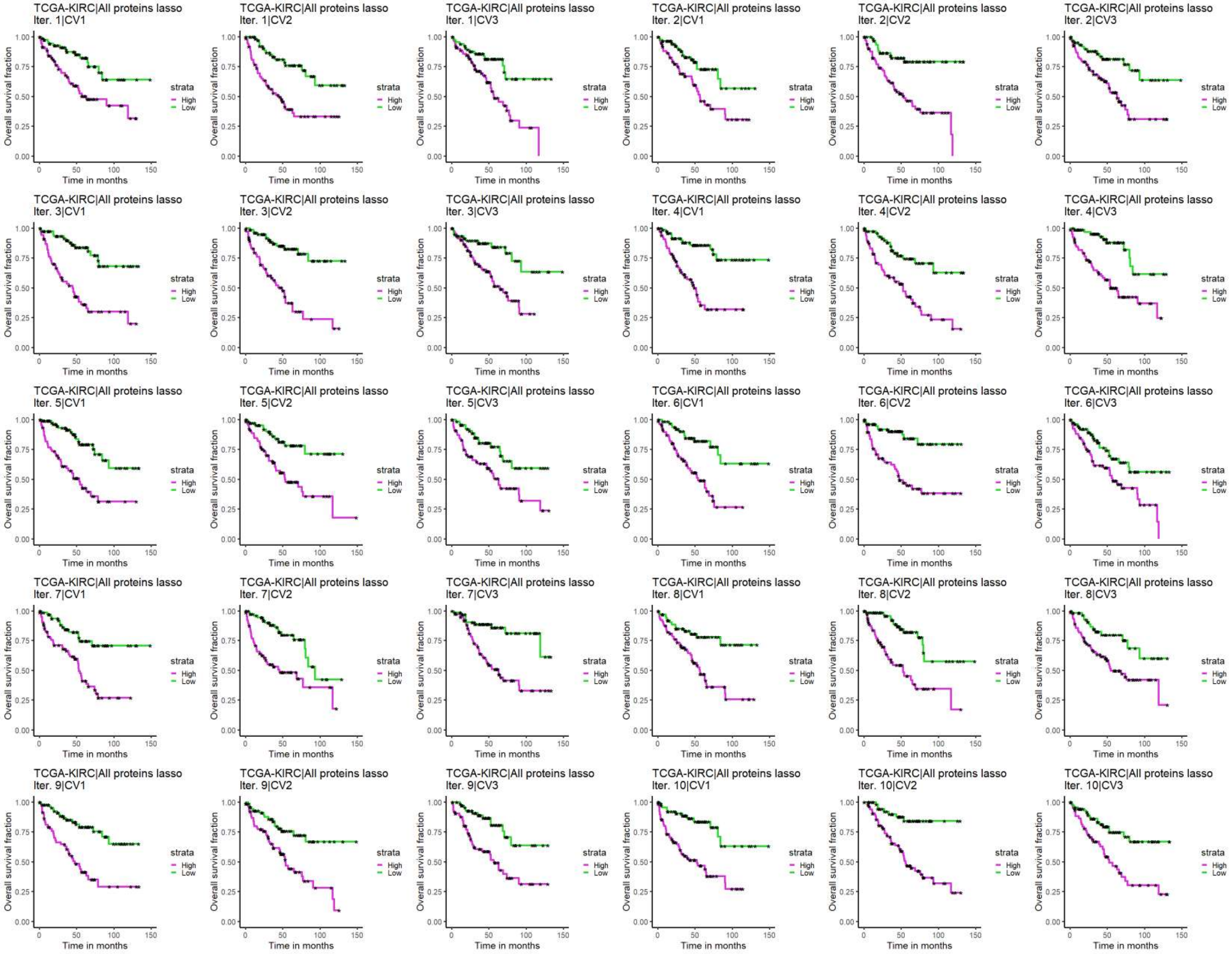

**Supplementary Figure S4.** Time-dependent receiver-operator characteristic curves (ROC; TP vs. FP) for 10 iterations of 3-fold cross-validation at five years of survival for **(A)** the unweighted RTK pathway score, **(B)** the Cox regression weighted RTK pathway score, and **(C)** the LASSO-derived protein signature score.

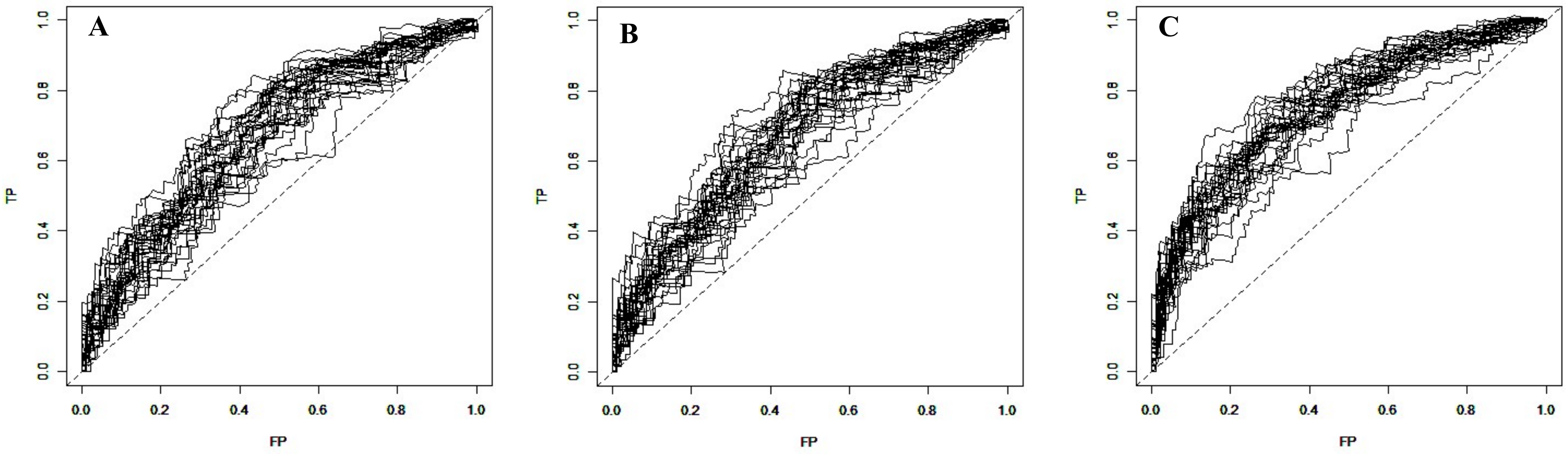

**Supplementary Figure S5.** Boxplots of risk scores by pathologic stage for **(A)** the original RTK score in KIRC, **(B)** the Cox-modified RTK score in KIRC, **(C)** the LASSO-derived risk score in KIRC, **(D)** the LASSO-derived risk score in OVCA, and **(E)** the LASSO-derived risk score in SKCM. In the SARC study, stage information was not provided in TCGA so the corresponding boxplot is not available. Stage X: unknown pathologic stage.

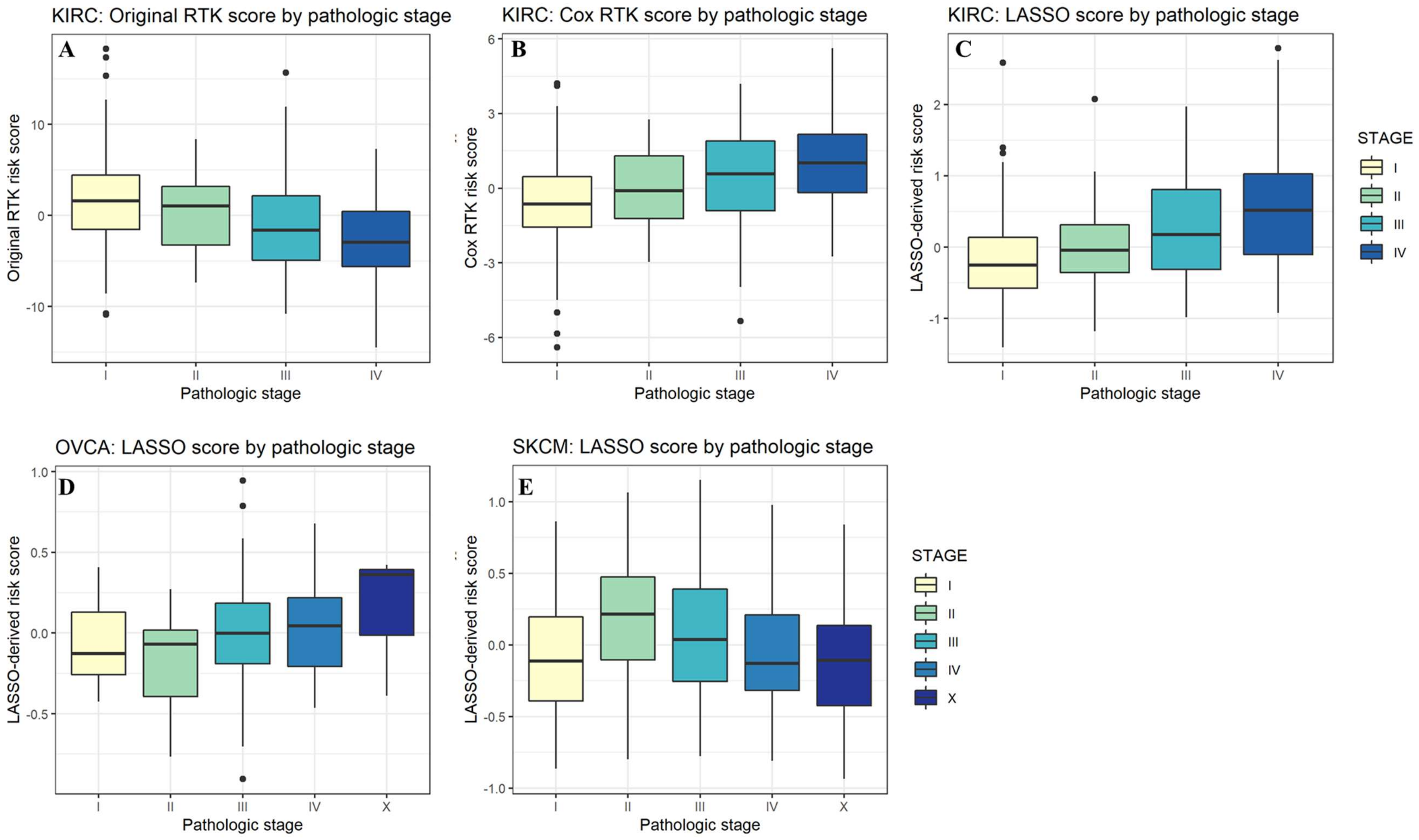

**Supplementary Figure S6.** Stage-separated and sex-separated Kaplan-Meier curves for **(A)** the original 7-protein RTK score, **(B)** the Cox-modified RTK score, and **(C)** the LASSO-derived risk score in KIRC. Ten-fold cross-validated risk scores were used.

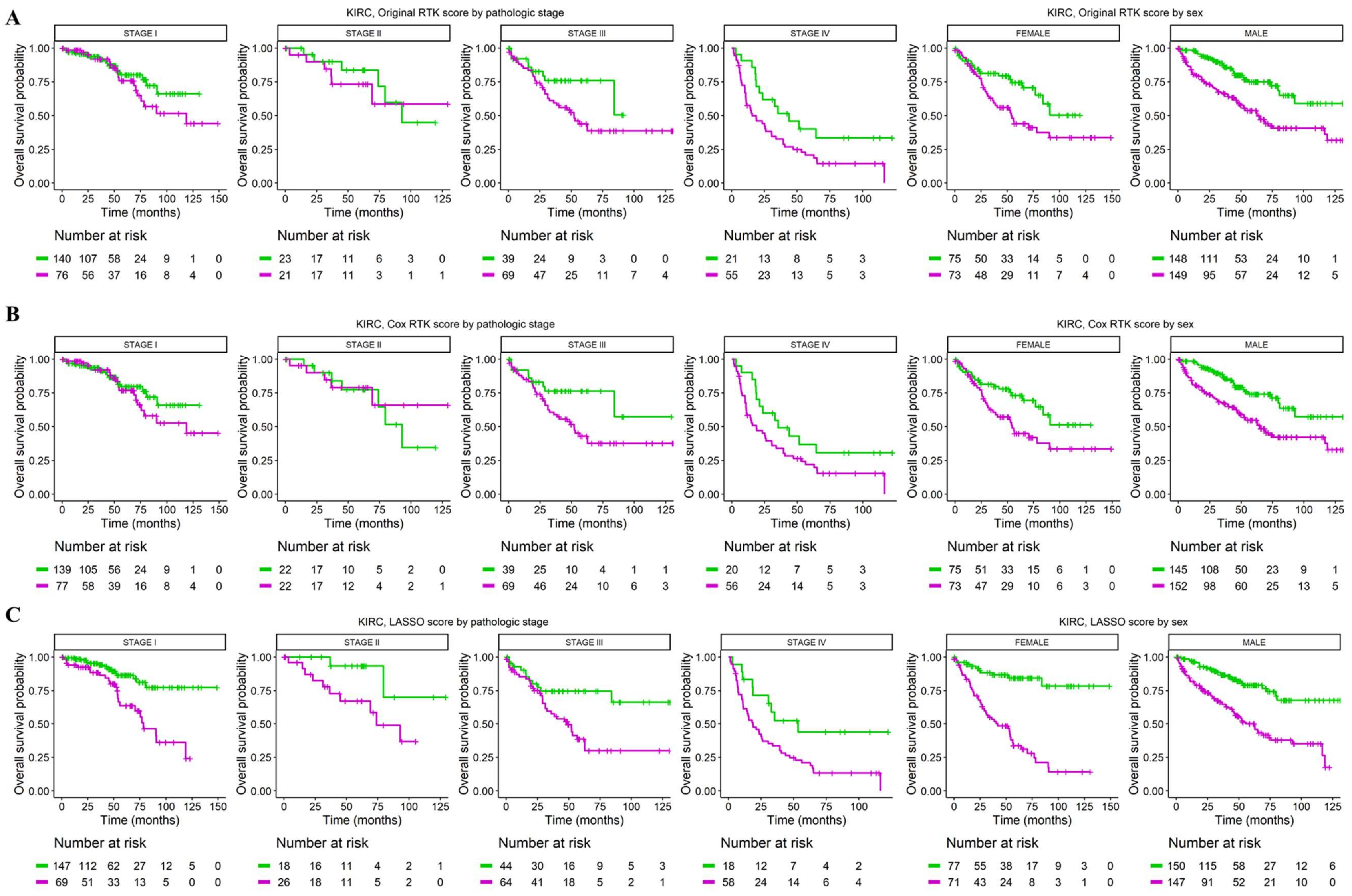

**Supplementary Figure S7.** LASSO Cox regression analysis for the identification of proteins associated with survival in **(A)** TCGA-SKCM, **(B)** TCGA-SARC, and **(C)** TCGA-OVCA. Ten-fold cross validation was conducted. The optimal value for tuning parameter λ and corresponding number of predictors with non-zero coefficients are indicated with the left-most dotted vertical line. The right-most vertical line denotes the largest λ such that the cross-validated error is within one standard error of the minimum. Predictors assigned non-zero coefficients for each dataset are included in the barplots. Proteins assigned to at least 1 the pre-defined pathways in (Akbani, *et al.*, 2014) are denoted as black bars with arrows. Proteins not in any of the 10 predefined pathways in the paper by Akbani *et al.* are shown as light grey bars.

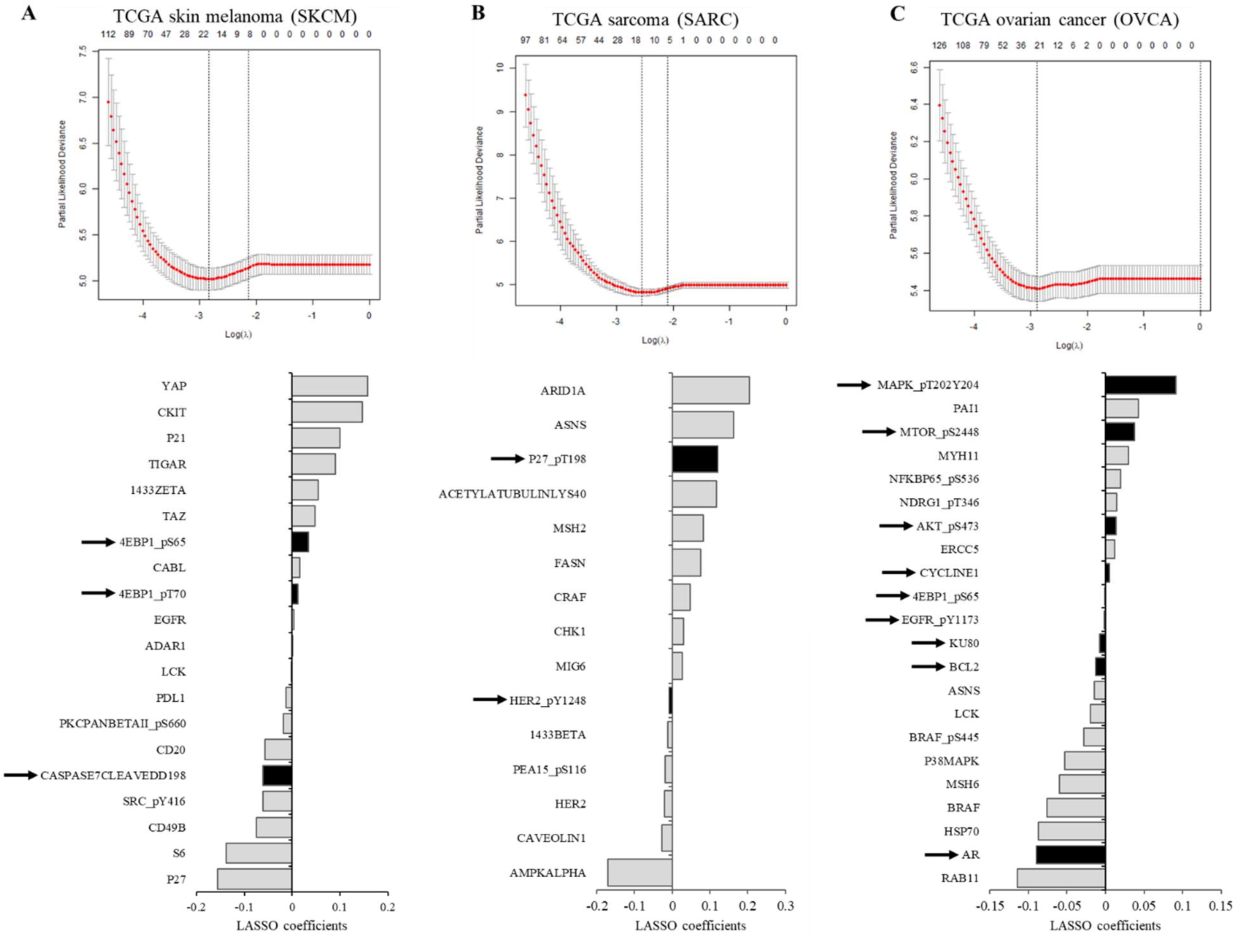

**Supplementary Figure S8.** Stage-separated and sex-separated Kaplan-Meier curves for the LASSO-derived risk score in **(A)** OVCA, **(B)** SKCM, and **(C)** SARC. Ten-fold cross-validated risk scores were used. In the OVCA study all patients were female and in the SARC study, stage information was not provided in TCGA so the corresponding plots are not available. Stage X: unknown pathologic stage**.**

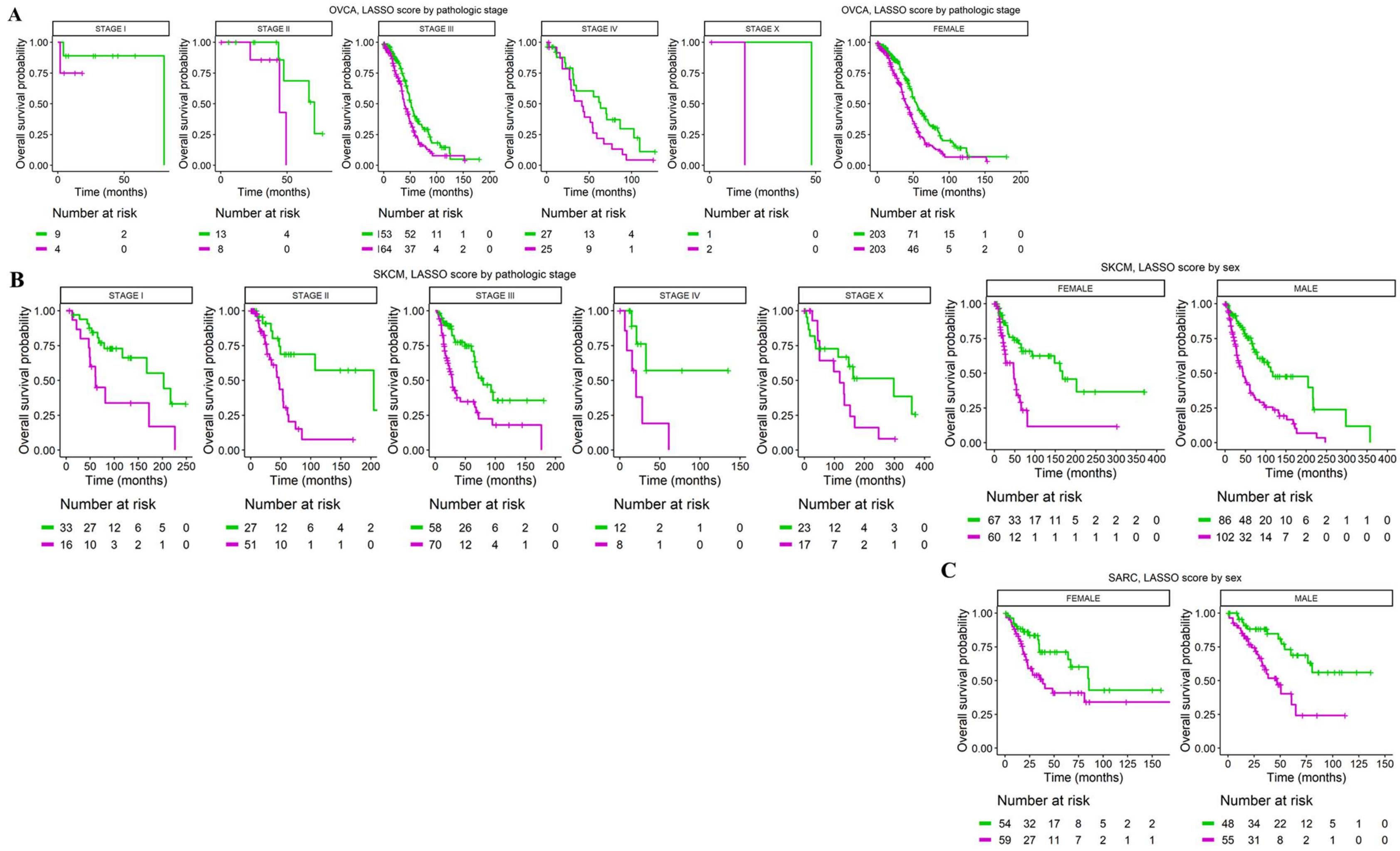

**Supplementary Table S1. Protein coefficient for literature-driven pathway score calculation.** Pathway predictors pre-defined in Akbani *et al.*, 2014 from a PubMed search of the literature. Positive regulators of a given pathway as described in relevant review papers were assigned a +1 coefficient, while negative regulators were assigned a −1 coefficient. The pathway score was defined as the unweighted sum of the median-centered, s.d. normalized protein expression value (i.e., relative protein level) of positive regulators minus the relative protein level of negative regulators.

| Pathway | Size | Literature-defined positive regulator (+1 coefficient) | Literature-defined negative regulator (−1 coefficient) |
|---|---|---|---|
| **Apoptosis** | 9 | BAK, BAX, BID, BIM, CASPASE7CLEAVEDD198 | BAD_pS112, BCL2, BCLXL, CIAP |
| **Cell_cycle** | 8 | CDK1, CYCLINB1, CYCLIND1, CYCLINE1, CYCLINE2, P27_pT157, P27_pT198, PCNA | |
| **DNA_damage_response** | 11 | 53BP1, ATM, BRCA2, CHK1_pS345, CHK2_pT68, KU80, MRE11, P53, RAD50, RAD51, XRCC1 | |
| **EMT** | 5 | COLLAGENVI, FIBRONECTIN, NCADHERIN | CLAUDIN7, ECADHERIN |
| **Hormone_a** | 3 | ERALPHA, ERALPHA_pS118, PR | |
| **Hormone_b** | 4 | AR, BCL2, GATA3, INPP4B | |
| **PI3K_AKT** | 10 | AKT_pS473, AKT_pT308, GSK3_pS9, GSK3ALPHABETA_pS21S9, P27_pT157, P27_pT198, PRAS40_pT246, TUBERIN_pT1462 | INPP4B, PTEN |
| **Ras_MAPK** | 10 | ARAF_pS299, CJUN_pS73, CRAF_pS338, JNK_pT183Y185, MAPK_pT202Y204, MEK1_pS217S221, P38_pT180Y182, P90RSK_pT359S363, SHC_pY317, YB1_pS102 | |
| **RTK** | 7 | EGFR_pY1068, EGFR_pY1173, HER2_pY1248, HER3_pY1289, SHC_pY317, SRC_pY416, SRC_pY527 | |
| **TSC_mTOR** | 8 | 4EBP1_pS65, 4EBP1_pT37T46, 4EBP1_pT70, MTOR_pS2448, P70S6K_pT389, RICTOR_pT1135, S6_pS235S236, S6_pS240S244 | |

**Supplementary Table S2.** Spearman correlation coefficients between the expression of the 7 RTK signature proteins from Akbani *et al.*, 2014 and the proteins most frequently selected by the LASSO in the TCGA-KIRC data (n=445 samples). Green denotes negative Spearman correlation coefficients while magenta denotes positive correlations.

| | ***7 RTK signature proteins*** | | | | | | |
|---|---|---|---|---|---|---|---|
| ***Proteins most frequently selected by the LASSO*** | *EGFR_ pY1068* | *EGFR_ pY1173* | *HER2_ pY1248* | *HER3_ pY1289* | *SHC_ pY317* | *SRC_ pY416* | *SRC_ pY527* |
| *4EBP1_pT37_T46* | 0.11 | -0.05 | -0.05 | -0.03 | 0.03 | -0.02 | 0 |
| *ACC1* | -0.22 | -0.26 | -0.32 | -0.2 | -0.35 | -0.12 | -0.35 |
| *AMPK-alpha_pT172* | 0.23 | 0.07 | 0.15 | 0.17 | 0.33 | 0.17 | 0.46 |
| *AR* | 0.33 | 0.09 | 0.21 | 0.18 | 0.36 | 0.22 | 0.37 |
| *A-Raf_pS299* | 0.07 | -0.01 | -0.09 | 0.01 | -0.01 | 0.09 | -0.06 |
| *B-Raf_pS445* | 0.12 | -0.16 | -0.15 | -0.09 | 0.02 | -0.04 | 0.15 |
| *Caveolin-1* | 0.08 | 0.12 | 0.08 | -0.11 | 0.01 | -0.02 | 0.13 |
| *CDK1* | -0.01 | 0.12 | 0.19 | 0.25 | 0.09 | 0.2 | -0.02 |
| *c-Myc* | -0.09 | 0.23 | 0.22 | 0.1 | -0.03 | -0.04 | -0.08 |
| *Gab2* | 0.2 | 0.27 | 0.27 | 0.1 | 0.23 | 0.12 | 0.21 |
| *IGFBP2* | -0.1 | 0.19 | 0.12 | 0.14 | -0.01 | 0.01 | -0.07 |
| *MAPK_pT202_Y204* | 0.37 | 0.23 | 0.42 | 0.48 | 0.72 | 0.53 | 0.55 |
| *MIG6* | 0.18 | 0.3 | 0.25 | 0.15 | 0.22 | 0.1 | 0.16 |
| *P70_S6K_pT389* | 0.13 | 0.17 | 0.24 | 0.52 | 0.36 | 0.24 | 0.11 |
| *PEA-15* | -0.18 | -0.07 | -0.22 | -0.35 | -0.3 | -0.33 | -0.2 |
| *Rad51* | -0.22 | 0.16 | 0.13 | 0.17 | -0.15 | -0.1 | -0.2 |
| *SCD1* | 0.06 | 0.09 | -0.06 | -0.06 | 0.06 | -0.12 | -0.09 |
| *SF2* | 0.07 | 0.19 | 0.07 | -0.06 | -0.04 | -0.06 | -0.1 |
| *Stat3_pY705* | 0.32 | 0.16 | 0.38 | 0.33 | 0.49 | 0.38 | 0.5 |
| *Syk* | -0.19 | -0.2 | -0.15 | -0.14 | -0.14 | 0.15 | -0.03 |
